# Theoretical and semi-analytical simulation for a two-predator-one-prey model during the mating period

**DOI:** 10.1371/journal.pone.0289410

**Published:** 2023-08-09

**Authors:** Noufe H. Aljahdaly

**Affiliations:** Mathematics Department, Faculty of Sciences and Arts, King Abdulaziz University, Rabigh, Saudi Arabia; Al al-Bayt University, JORDAN

## Abstract

The article introduces a new application which is a system of equations of two predators and one prey with the term of interaction between male and female of predators and prey. Such term appears when male and female of predators feed on the same prey during their mating period. The mathematical model has been studied theoretically and semi-analytically. The positivity, boundedness, local and global stability are proved for the system. The logarithm of multistage differential transform method (MsDTM) is used to study this new application. The MsDTM is used because it globally converges to the solution, it is a highly accurate, fast and simple approach. The stability analysis as well as semi-analytical solutions of the system are obtained to understand the dynamic of the model. Moreover, the effects of several parameters in the system are presented. As a results, we obtain the periodic solution when when the growth rate of prey is larger than the growth rate of both type of predators.

## 1 Introduction

Mathematical models phrased in terms of differential equations are very useful tools to describe several problems and applications in science such as biology [1], physics [2], fluid dynamic [3], medical science [4, 5], etc. They are helpful to control the spread of infection, to understand the behavior of population, to observe the effect of several factors in population or to predict the solutions for different problems in the world. Some populations are described by a system of ordinary differential equations (ODEs) that introduces the interaction of different species [6].

The predator-prey model is a well known mathematical model and it was first introduced by Lotka [7] and Volterra [8]. It has been used widely to study the dynamic of interaction of two species in population ecology and associated ecological interactions [9]. In addition, researchers modified the model to study different biological and ecological scenarios. For example, they study the model among vertebrates and invertebrates in freshwater systems associated with fisheries [10] and with prey refuge and delay [11]. The modified models have been utilized for studying the impulsive effects [12], the impact of prey herd dread from the two types of predators [13], effect of food sharing among predators [14], the effect of two different food chains [15], studying the stationary probability distributions of its population densities [16], studying with disease in prey [17] and so on.

One of the behaviors of some type of animals such as the discus fish is that the male stays with the female during mating period to protect her. During this period both male and female feed together in same prey. The model that describes the interaction between one predator and two preys has been studied in [18, 19], while the model of two predators and one prey has been studied in a few papers such as [20, 21]. Al Qudah modified the predator-prey model by considering the effect of the term *XY*^2^, where *Y*^2^ denotes the intersection between a male and a female of predators who feed on same prey (*X*) during mating period [22]. Then, Aljahdaly and Al qudah found the analytical solutions and discussed the biological relevance of the solutions [23]. The numerical solution was found by the exponential time differencing method [24]. However, no contributions in the existing literature have studied the effect of the term *XY*^2^ on the two-predators-one-prey model.

Due to the difficulty of computing exact solutions for most ODEs that described real applications, semi-analytical solutions are found instead of exact solutions in explicit form [25]. Our previous work [26] presented the predator-prey with the effect of the term *XY*^2^ and computed the semi-analytical solution by the differential transform method (DTM) [27], Adomian decomposition method (ADM) [28], 4^th^ Runge-Kutta (4^th^RK) [29] and the multistage differential transform method (MsDTM). We proved that the MsDTM is more accurate than DTM and ADM and faster than the 4^th^RK. In fact, semi-analytical methods find the solution in the form of series and the other terms of series are obtained from given initial conditions. Some semi-analytical techniques are based on Taylor expansion about the initial point *t*_0_ such as Legendre Pseudospectral Scheme [30], residual power series method (RPSM) Laplace decomposition method (LDM) [31], DTM and ADM. These methods enable to find the solution in a very small time domain because the Taylor expansion is locally convergent. In other words, the DTM can find the solution only in small neighborhood about the initial point and can not obtain the solution in long domain. The DTM has been improved by using Padé approximation and by applying the multistage technique. Both improvements are able to find the solutions in long domain. However, the MsDTM is a reliable and optimal method to approximate solutions in a long domain for linear and nonlinear ODEs [26]. The MsDTM is a modification of the DTM by dividing the original time domain into sub-domains and applying the DTM method at each subdomain. In addition, the nonlinearity is treated by transforming the nonlinear term to a special summation based on differential transformation form. The accuracy of the MsDTM is controlled by the iteration number and time step size. The accuracy for the 4^th^RK is controlled only by the time step size, therefore, the MsDTM can be faster than 4^th^RK [32].

The novelty of this paper is presenting a two-predator-one-prey mathematical model during the predator mating period. In addition the paper studies the model theoretically including a positivity, boundedness, as well as local and global stability. The paper introduces the MsDTM logarithm and interprets the results.

The paper is organized as follows: section (2) proposes the mathematical model, section (3) shows the positivity and boundedness, section (4) studies the local and global stability, section (4) describes the MsDTM for finding the semi-analytical solutions and these results are discussed in section (6), and the last section contains a conclusion of this work.

## 2 Mathematical model

In this section, we introduce the mathematical model that describes the interactions between two types of predators (i.e *Y* and *Z*) and one type of prey (*X*). We aim to study the behavior of the animal population during the mating period under the following assumptions:

- a male of the predators stays with the same female and they feed on the same prey during their mating period.
- the rate of growth for predators and prey are larger than the rate of decay by their natural death.

The modified diffusive predator-prey model in reference [22] studied the interaction of two predators (male and female from the same species) with one prey in the mating period. Herein, we extend this model to study two species of predators interacting with one prey during their mating period as follows

$$\begin{array}{cc} X^{\prime }= & \beta_{1}X-\beta_{2}X-\beta_{3}X^{2}-\beta_{4}XY-\beta_{5}XY^{2}-\beta_{6}XZ^{2}-\beta_{7}XZ,\\ Y^{\prime }= & \beta_{8}Y+\beta_{4}XY-\beta_{9}Y-\beta_{10}Y^{2}+\beta_{5}XY^{2},\\ Z^{\prime }= & \beta_{11}Z+\beta_{7}XZ-\beta_{12}Z-\beta_{13}Z^{2}+\beta_{6}XZ^{2}, \end{array}$$ (1)

where *X*(*t*), *Y*(*t*) and *Z*(*t*) denote densities of prey, predator type (I) and predator type (II), respectively. The terms *XY* and *XZ* refer to the interaction between prey and predators of type (I) and type (II) respectively. The terms *XY*^2^ and *XZ*^2^ represent the interaction between the male and female of predators and prey. All parameters are nonnegative and have the following interpretations

- *β*_1_, *β*_8_, *β*_11_ are the growth rates of *X*, *Y*, *Z* respectively.
- *β*_2_, *β*_9_, *β*_12_ are the decay rates of *X*, *Y*, *Z* respectively due to natural death.
- *β*_3_, *β*_10_, *β*_13_ are the decay rates of *X*, *Y*, *Z* respectively due to competition of food on the same species.
- *β*_4_, *β*_7_ are the decay rates of prey due to the interaction between one predator and one prey.
- *β*_5_, *β*_6_ are the decay rates of prey due to the interaction between two predators and one prey.

## 3 Positivity and boundedness

**Theorem 1** *All solutions of system* (1) *are nonnegative and bounded in some region* Ω *subject to nonnegative initial conditions in* Ω.

**Proof**

Let assume the initial conditions at *t* = 0 are nonnegative and rewrite the system (1) as

$$\frac{X^{\prime }\left(t\right)}{X(t)}=U\left(t\right),\;\frac{Y^{\prime }\left(t\right)}{Y(t)}=V\left(t\right),\;\frac{Z^{\prime }\left(t\right)}{Z(t)}=W\left(t\right)$$

where

$$\begin{array}{cc} U(t)≕ & \beta_{1}-\beta_{2}-\beta_{3}X\left(t\right)-\beta_{4}Y\left(t\right)-\beta_{5}Y{\left(t\right)}^{2}-\beta_{6}Z{\left(t\right)}^{2}-\beta_{7}Z\left(t\right),\\ V(t)≕ & \beta_{8}+\beta_{4}X\left(t\right)-\beta_{9}-\beta_{10}Y\left(t\right)+\beta_{5}X\left(t\right)Y\left(t\right),\;\text{and}\\ W(t)≕ & \beta_{11}+\beta_{7}X\left(t\right)-\beta_{12}-\beta_{13}Z\left(t\right)+\beta_{6}X\left(t\right)Z\left(t\right). \end{array}$$

by integrating over [0, *t*], the following equalities are valid:

$$X\left(t\right)=X\left(0\right)\;\text{exp}\;(\int_{0}^{t}U\left(\rho \right)d\rho ),$$

$$Y\left(t\right)=Y\left(0\right)\;\text{exp}\;(\int_{0}^{t}V\left(\rho \right)d\rho ),$$

and

$$Z\left(t\right)=Z\left(0\right)\;\text{exp}\;(\int_{0}^{t}W\left(\rho \right)d\rho ).$$

Since *X*(0) ≥ 0,*Y*(0) ≥ 0, and *Z*(0) ≥ 0, we have *X*(*t*) ≥ 0, *Y*(*t*) ≥ 0 and *Z*(*t*) ≥ 0 for all *t* ≥ 0. Hence, the solution (X(t),Y(t), Z(t)) is nonnegative for nonnegative initial data. Next, we prove that all solutions will remain bounded. We start by rewriting the system (1) as follows

$$\begin{array}{cc} X^{\prime }+Y^{\prime }+Z^{\prime } & =\left(\beta_{1}-\beta_{2}\right)X-\beta_{3}X^{2}+\left(\beta_{3}-\beta_{9}\right)Y-\beta_{10}Y^{2}+\left(\beta_{11}-\beta_{12}\right)Y-\beta_{13}Z^{2}\\ & \leq \gamma_{1}\left(X+Y+Z\right)-\gamma_{2}\left(X^{2}+Y^{2}+Z^{2}\right) \end{array}$$

Let *β*_1_ > *β*_2_, *β*_3_ > *β*_9_, *β*_11_ > *β*_12_, *γ*_1_ = max{(*β*_1_ − *β*_2_), (*β*_3_ − *β*_9_), (*β*_11_ − *β*_12_)}, and *γ*_2_ = min{*β*_3_, *β*_10_, *β*_13_}, thus,

$$\text{lim}\;\underset{t\to \infty }{\text{sup}}\;\left(\gamma_{1}\left(X+Y+Z\right)-\gamma_{2}\left(X^{2}+Y^{2}+Z^{2}\right)\right)\leq 0.$$

Hence, all the solutions of model (1) are nonnegative and bounded in the following region

$$\Omega =\{\left(X,Y,Z\right)\in R_{3}^{+}:0\leq X,Y,Z\leq \frac{\gamma_{1}}{\sqrt{\gamma_{2}}}\}.$$

## 4 Stability analysis

An equilibrium point of a dynamical system denotes a stationary condition for the dynamics. The local stability at equilibrium points indicates that the solutions remain near the equilibrium points with small perturbation, but can lead to a large change with large perturbation. On the other hand, the global stability means the solution remain nearby the equilibrium points with small or large perturbations. Thus, in this section, we will study the local and global stability of the equilibrium points.

### 4.1 Equilibrium points

To study the stability of the system (1), the equilibrium points are obtained by setting

$$\begin{array}{c} F_{1}\left(X,Y,Z\right)≕\beta_{1}X-\beta_{2}X-\beta_{3}X^{2}-\beta_{4}XY-\beta_{5}XY^{2}-\beta_{6}XZ^{2}-\beta_{7}XZ=0,\\ F_{2}\left(X,Y,Z\right)≕\beta_{8}Y+\beta_{4}XY-\beta_{9}Y-\beta_{10}Y^{2}+\beta_{5}XY^{2}=0.\\ F_{3}\left(X,Y,Z\right)≕\beta_{11}Z+\beta_{7}XZ-\beta_{12}Z-\beta_{13}Z^{2}+\beta_{6}XZ^{2}=0. \end{array}$$ (2)

The solutions for the system (2) give the equilibrium points as follows: $P_{1}=\left(0,\frac{\beta_{8}-\beta_{9}}{\beta_{10}},\frac{\beta_{11}-\beta_{12}}{\beta_{13}}\right),\;P_{2}=\left(0,0,\frac{\beta_{11}-\beta_{12}}{\beta_{13}}\right),\;P_{3}=\left(0,\frac{\beta_{8}-\beta_{9}}{\beta_{10}},0\right)$ and $P_{4}=\left(\frac{\beta_{1}-\beta_{2}}{\beta_{3}},0,0\right)$. Note that the coexisting equilibrium point were excluded because it is negative points. Also, the trivial point was excluded because is not interesting in our investigation.

### 4.2 Local stability

**Theorem 2** *The points*, $\left(0,\frac{\beta_{8}-\beta_{9}}{\beta_{10}},\frac{\beta_{11}-\beta_{12}}{\beta_{13}}\right),\left(0,0,\frac{\beta_{11}-\beta_{12}}{\beta_{13}}\right),\left(0,\frac{\beta_{8}-\beta_{9}}{\beta_{10}},0\right)$ *and* $\left(\frac{\beta_{1}-\beta_{2}}{\beta_{3}},0,0\right)$ *are locally asymptotically stable if the following conditions are satisfied*

- *(1): for the equilibrium point*: $\left(0,\frac{\beta_{8}-\beta_{9}}{\beta_{10}},\frac{\beta_{11}-\beta_{12}}{\beta_{13}}\right)$
  *Local stability conditions*: *β*_8_ ≥ *β*_9_, *β*_11_ ≥ *β*_12_, $\beta_{1}\leq \frac{\beta_{6}(\beta_{11}-\beta_{12}){}^{2}+(\beta_{7}(\beta_{11}-\beta_{12})+\frac{(\beta_{5}(\beta_{8}-\beta_{9}){}^{2}+\beta_{10}(\beta_{4}(\beta_{8}-\beta_{9})+\beta_{2}\beta_{10}))\beta_{13}}{\beta_{10}^{2}})\beta_{13}}{\beta_{13}^{2}},\;\beta_{10}\neq 0,\beta_{13}\neq 0,$
- *(2): for the equilibrium point*: $\left(0,0,\frac{\beta_{11}-\beta_{12}}{\beta_{13}}\right)$
  *Local stability conditions*: $\beta_{8}\leq \beta_{9},\;\beta_{11}\geq \beta_{12},\;\beta_{1}\leq \frac{\beta_{6}(\beta_{11}-\beta_{12}){}^{2}+\beta_{13}(\beta_{7}(\beta_{11}-\beta_{12})+\beta_{2}\beta_{13})}{\beta_{13}^{2}},\;\beta_{13}\neq 0,$
- *(3): for the equilibrium point*: $\left(0,\frac{\beta_{8}-\beta_{9}}{\beta_{10}},0\right)$
  *Local stability conditions*: $\beta_{8}\geq \beta_{9},\;\beta_{11}\leq \beta_{12},\;\beta_{1}\leq \frac{\beta_{5}(\beta_{8}-\beta_{9}){}^{2}+\beta_{10}(\beta_{4}(\beta_{8}-\beta_{9})+\beta_{2}\beta_{10})}{\beta_{10}^{2}},\;\beta_{10}\neq 0,$
- *(4): for the equilibrium point*: $\left(\frac{\beta_{1}-\beta_{2}}{\beta_{3}},0,0\right)$
  *Local stability conditions*: $\beta_{1}\geq \beta_{2},\beta_{9}\geq \frac{\beta_{1}\beta_{4}}{\beta_{3}}-\frac{\beta_{2}\beta_{4}}{\beta_{3}}+\beta_{8},\beta_{12}\geq \frac{\beta_{1}\beta_{7}}{\beta_{3}}-\frac{\beta_{2}\beta_{7}}{\beta_{3}}+\beta_{11},\;\;\beta_{3}\neq 0.$

**Proof**

The Jacobian matrix for the system (1) is defined as

$$J\left(X,Y,Z\right)=[\begin{array}{ccc} \frac{\partial F_{1}}{\partial X} & \frac{\partial F_{1}}{\partial Y} & \frac{\partial F_{1}}{\partial Z}\\ \frac{\partial F_{2}}{\partial X} & \frac{\partial F_{2}}{\partial Y} & \frac{\partial F_{2}}{\partial Z}\\ \frac{\partial F_{3}}{\partial X} & \frac{\partial F_{3}}{\partial Y} & \frac{\partial F_{3}}{\partial Z} \end{array}],$$

$$J=(\begin{array}{ccc} A_{1} & \beta_{4}\left(-X\right)-2\beta_{5}XY & -\beta_{7}X-2\beta_{6}XZ\\ \beta_{5}Y^{2}+\beta_{4}Y & A_{2} & 0\\ \beta_{6}Z^{2}+\beta_{7}Z & 0 & A_{3} \end{array}),$$

where *A*_1_ = *β*_1_ − *β*_2_ − 2*β*_3_*X* − *β*_5_*Y*^2^ − *β*_4_*Y* − *β*_6_*Z*^2^ − *β*_7_*Z*, *A*_2_ = *β*_8_ − *β*_9_ + *β*_4_*X* + 2*β*_5_*XY* − 2*β*_10_*Y*, *A*_3_ = *β*_11_ − *β*_12_ + *β*_7_*X* + 2*β*_6_*XZ* − 2*β*_13_*Z*. To study the stability of each equilibrium point, we substitute the point *P*_*i*_, *i* = 1, 2, 3, 4 into the matrix *J* and the result is *J*_*P*_. Then, looking for negative eigenvalues of *J*_*P*_ implies the stability conditions for each equilibrium point.

(1): the equilibrium point: $\left(0,\frac{\beta_{8}-\beta_{9}}{\beta_{10}},\frac{\beta_{11}-\beta_{12}}{\beta_{13}}\right)$ is non negative if *β*_8_ ≥ *β*_9_, *β*_11_ ≥ *β*_12_, and the eigenvalue of $J_{P_{1}}$ is negative if $\beta_{1}\leq \frac{\beta_{6}(\beta_{11}-\beta_{12}){}^{2}+(\beta_{7}(\beta_{11}-\beta_{12})+\frac{(\beta_{5}(\beta_{8}-\beta_{9}){}^{2}+\beta_{10}(\beta_{4}(\beta_{8}-\beta_{9})+\beta_{2}\beta_{10}))\beta_{13}}{\beta_{10}^{2}})\beta_{13}}{\beta_{13}^{2}},\;\beta_{10}\neq 0,\beta_{13}\neq 0$
(2): $P_{2}=\left(0,0,\frac{\beta_{11}-\beta_{12}}{\beta_{13}}\right)$ is non-negative if *β*_8_ ≤ *β*_9_, *β*_11_ ≥ *β*_12_ and the eigenvalues of $J_{P_{2}}$ are negative if $\beta_{1}\leq \frac{\beta_{6}(\beta_{11}-\beta_{12}){}^{2}+\beta_{13}(\beta_{7}(\beta_{11}-\beta_{12})+\beta_{2}\beta_{13})}{\beta_{13}^{2}},\;\beta_{13}\neq 0$
(3): $P_{3}=\left(0,\frac{\beta_{8}-\beta_{9}}{\beta_{10}},0\right)$ is non-negative if *β*_8_ ≥ *β*_9_, *β*_11_ ≤ *β*_12_, and the eigenvalues of $J_{P_{3}}$ are negative if $\;\beta_{1}\leq \frac{\beta_{5}(\beta_{8}-\beta_{9}){}^{2}+\beta_{10}(\beta_{4}(\beta_{8}-\beta_{9})+\beta_{2}\beta_{10})}{\beta_{10}^{2}},\;\beta_{10}\neq 0$
(4): $P_{4}=\left(\frac{\beta_{1}-\beta_{2}}{\beta_{3}},0,0\right)$ is non- negative if *β*_1_ ≥ *β*_2_, and the eigenvalues of $J_{P_{4}}$ are negative if $\beta_{9}\geq \frac{\beta_{1}\beta_{4}}{\beta_{3}}-\frac{\beta_{2}\beta_{4}}{\beta_{3}}+\beta_{8},\beta_{12}\geq \frac{\beta_{1}\beta_{7}}{\beta_{3}}-\frac{\beta_{2}\beta_{7}}{\beta_{3}}+\beta_{11},\;\;\beta_{3}\neq 0$

Thus, the points, $\left(0,\frac{\beta_{8}-\beta_{9}}{\beta_{10}},\frac{\beta_{11}-\beta_{12}}{\beta_{13}}\right),\left(0,0,\frac{\beta_{11}-\beta_{12}}{\beta_{13}}\right),\left(0,\frac{\beta_{8}-\beta_{9}}{\beta_{10}},0\right)$ and $\left(0,0,\frac{\beta_{11}-\beta_{12}}{\beta_{13}}\right)$ are asymptotically stable associated to the presented stability conditions. The relevant ecological interpretation for stability analysis that the system has stable equilibrium point (1) if the growth rate of predators is greater than the natural death rate for both types of predators. The equilibrium point (2) is stable if the growth rate of the predator type (I) is less than the death rate and the growth rate of predator type (II) is greater than its death rate. In case of the opposite of this scenario, the equilibrium point (3) is stable. The equilibrium point (4) is stable if the growth rate of the prey is greater than the death rate which is a result of decreasing growth rate of predators type (I) and (II).

### 4.3 Global stability

**Theorem 3** *The equilibrium points are asymptotically global stable under the following stability conditions*

*(1) for point* $\left(0,\frac{\beta_{8}-\beta_{9}}{\beta_{10}},\frac{\beta_{11}-\beta_{12}}{\beta_{13}}\right)$ , *the conditions are β*_8_ ≥ *β*_9_, *β*_11_ ≥ *β*_12_, *β*_10_ ≠ 0, *β*_13_ ≠ 0 *and* *β*_1_ ≤ *β*_2_,
*(2) for point* $\left(0,0,\frac{\beta_{11}-\beta_{12}}{\beta_{13}}\right)$ , *the conditions are β*_11_ ≥ *β*_12_, *β*_13_ ≠ 0, *β*_1_ ≤ *β*_2_, *β*_8_ ≤ *β*_9_,
*(3) for point* $\left(0,\frac{\beta_{8}-\beta_{9}}{\beta_{10}},0\right)$ , *the conditions are β*_8_ ≥ *β*_9_, *β*_10_ ≠ 0, *β*_1_ ≤ *β*_2_, *β*_11_ ≤ *β*_12_,
*(4) for point* $\left(\frac{\beta_{1}-\beta_{2}}{\beta_{3}},0,0\right)$ , *the conditions are β*_1_ ≥ *β*_1_, *β*_3_ ≠ 0, *β*_8_ ≤ *β*_9_, *β*_11_ ≤ *β*_12_, *and* *β*_5_ = *β*_4_ = *β*_6_ = *β*_7_ = 0.

**Proof**

The global stability can be studied by employing the Lyapunov equation. Consider

$$V\left(X,Y,Z\right)=V_{1}\left(X,Y,Z\right)+V_{2}\left(X,Y,Z\right)+V_{3}\left(X,Y,Z\right),$$

where

$$\begin{array}{c} V_{1}\left(X,Y,Z\right)=X-X^{*}-X^{*}\;\text{ln}\left(\frac{X}{X^{*}}\right),\\ V_{2}\left(X,Y,Z\right)=Y-Y^{*}-Y^{*}\;\text{ln}\left(\frac{Y}{Y^{*}}\right),\\ V_{3}\left(X,Y,Z\right)=Z-Z^{*}-Z^{*}\;\text{ln}\left(\frac{Z}{Z^{*}}\right). \end{array}$$

These functions are defined on $R_{+}^{3}$ and (X*, Y*, Z*) is an equilibrium point. The function *V*(*X*, *Y*, *Z*) is zero at all equilibrium points and positive for other values. An equilibrium point *p* is globally stable if the derivative $\frac{dV}{dt}$ evaluated at *p* is non-positive, that is if $\frac{dV}{dt}\left(p\right)\leq 0$.

- for point $P_{1}=\left(0,Y_{1}^{*},Z_{1}^{*}\right)$, the point is positive defined under the conditions *β*_8_ ≥ *β*_9_, *β*_11_ ≥ *β*_12_, *β*_10_ ≠ 0, *β*_13_ ≠ 0 and from the equilibrium equations we have
  $$\beta_{8}=\beta_{9}+\beta_{10}Y_{1}^{*},\;\;\beta_{11}=\beta_{12}+\beta_{13}Z_{1}^{*},$$
  $$\begin{array}{cc} \frac{dV}{dt}= & -X\left(t\right)(-\beta_{1}+\beta_{2}+\beta_{3}X\left(t\right)+\beta_{5}Y_{1}^{*}Y\left(t\right)+\beta_{6}Z_{1}^{*}Z\left(t\right)+\beta_{4}Y_{1}^{*}+\beta_{7}Z_{1}^{*})\\ & -\beta_{10}{\left(Y_{1}^{*}-Y\left(t\right)\right)}^{2}-\beta_{13}{\left(Z_{1}^{*}-Z\left(t\right)\right)}^{2}, \end{array}$$
  $\frac{dV}{dt}<0$, if *β*_1_ ≤ *β*_2_.
- for point $P_{2}=\left(0,0,Z_{2}^{*}\right)$, the point is positive defined under the conditions *β*_11_ ≥ *β*_12_, *β*_13_ ≠ 0 and from the equilibrium equations we have
  $$\beta_{11}=\beta_{12}+\beta_{13}Z_{2}^{*},$$
  $$\begin{array}{cc} \frac{dV}{dt}= & -X\left(t\right)(-\beta_{1}+\beta_{2}+\beta_{3}X\left(t\right)+\beta_{6}Z_{2}^{*}Z\left(t\right)+\beta_{7}Z_{2}^{*})-\beta_{10}Y{\left(t\right)}^{2}+Y\left(t\right)(\beta_{8}-\beta_{9})\\ & -\beta_{13}{\left(Z_{2}^{*}-Z\left(t\right)\right)}^{2}, \end{array}$$
  $\frac{dV}{dt}<0$, if *β*_1_ ≤ *β*_2_, *β*_8_ ≤ *β*_9_.
- for point $P_{3}=\left(0,Y_{3}^{*},0\right)$, the point is positive defined under the conditions *β*_8_ ≥ *β*_9_, *β*_10_ ≠ 0 and from the equilibrium equations we have
  $$\beta_{8}=\beta_{9}+\beta_{10}Z_{2}^{*},$$
  $$\begin{array}{c} \frac{dV}{dt}=-X\left(t\right)(-\beta_{1}+\beta_{2}+\beta_{3}X\left(t\right)+\beta_{5}Y{\left(t\right)}^{2}+\beta_{4}Y\left(t\right))-\beta_{13}Z{\left(t\right)}^{2}+Z\left(t\right)(\beta_{11}-\beta_{12}), \end{array}$$
  $\frac{dV}{dt}<0$, if *β*_1_ ≤ *β*_2_, *β*_11_ ≤ *β*_12_.
- for point $P_{4}=\left(X_{4}^{*},0,0\right)$, the point is positive defined under the conditions *β*_1_ ≥ *β*_1_, *β*_3_ ≠ 0 and from the equilibrium equations we have
  $$\beta_{1}=\beta_{2}+\beta_{3}X_{4}^{*},$$
  $$\begin{array}{cc} \frac{dV}{dt}= & -\beta_{3}{\left(X_{4}^{*}-X\left(t\right)\right)}^{2}+X_{4}^{*}Y\left(t\right)(\beta_{5}Y\left(t\right)+\beta_{4})+X_{4}^{*}Z\left(t\right)(\beta_{6}Z\left(t\right)+\beta_{7})-\beta_{10}Y{\left(t\right)}^{2}+\\ & Y\left(t\right)(\beta_{8}-\beta_{9})-\beta_{13}Z{\left(t\right)}^{2}+Z\left(t\right)(\beta_{11}-\beta_{12}), \end{array}$$
  $\frac{dV}{dt}<0$, if *β*_8_ ≤ *β*_9_, *β*_11_ ≤ *β*_12_, and *β*_5_ = *β*_4_ = *β*_6_ = *β*_7_ = 0.

## 5 The multistage differential transform method

The idea of the multistage differential transform method is based on dividing the domain of time into subdomains [*t*_*i*_ *t*_*i*+1_]. In each subdomain, we apply the differential transform method (DTM) where the initial condition at *t*_*i*_. The DT is defined as follows

$$\begin{array}{c} U\left(k\right)=\frac{1}{k!}\frac{d^{k}u\left(t\right)}{dt^{k}}{|}_{t=t_{0}}. \end{array}$$ (3)

Let assume an ODE with initial condition at *t*_0_ = 0 and a solution *u*(*t*). The operators in the ODE are transferred to DT operators as in Table 1. Then, we obtain the scheme in terms of *U*(*k*) and *U*(*k* + 1). The *U*(0) is the initial condition and *U*(*k* + 1) is obtained by applying *U*(*k*). The solution *u*(*t*) is $\sum_{k=0}^{N}U\left(k\right){\left(t-t_{i}\right)}^{k}$, where *N* is the arbitrary iteration number. The readers are referred to the references [26] for more details.

**Table 1:** Differential transformations.

Original function	DT function
*u*(*t*) + *υ*(*t*)	*U*(*k*) + *V*(*k*)
*cu*(*t*)	*cU*(*k*)
*u*(*t*)*υ*(*t*)	$\sum_{p=0}^{k}U\left(p\right)V\left(k-p\right)$
$\frac{du(t)}{dt}$	(*k* + 1)*U*(*k* + 1)
$\frac{d^{2}u\left(t\right)}{dt^{2}}$	(*k* + 1)(*k* + 2)*U*(*k* + 2)
$\frac{d^{m}u\left(t\right)}{dt^{m}}$	$\frac{(k+m)!}{m!}U\left(k+m\right)$
*t* ^*n*^	$\delta \left(k-n\right)=\{\begin{array}{cc} 1 & \;\text{k = n}\;\\ 0 & \;\text{k}\neq \;\text{n} \end{array}$

## 6 Semi-analytical simulation

**Fig 1:**
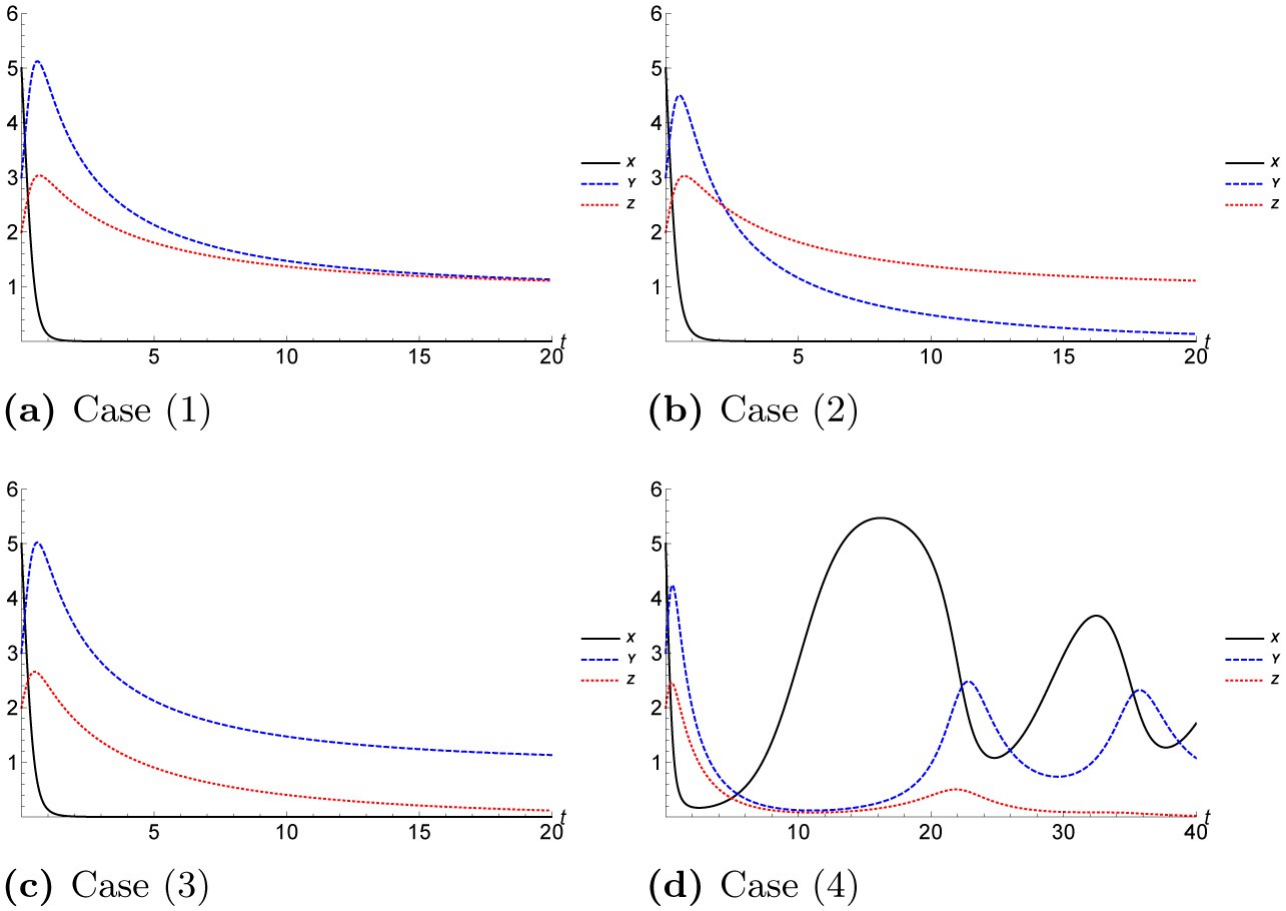
Plot the solutions of *X*(*t*), *Y*(*t*) and *Z*(*t*) where *h* = 0.01 using MsDTM for *X*_0_ = 5, *Y*_0_ = 3 and *Z*_0_ = 2. (a) Case (1), (b) Case (2), (c) Case (3), (d) Case (4).

In this section, the proposed model will be computed by MsDTM. First step, we divide the domain [0, *T*] into subdomain [*t*_*i*_, *t*_*i*+1_] where *h* = [*t*_*i*+1_ − *t*_*i*_]/100 is the step size. Next, we assume $X=\sum_{k=0}^{\alpha }X^{k}t^{k},\;Y=\sum_{k=0}^{\alpha }Y^{k}t^{k},\;Z=\sum_{k=0}^{\alpha }Z^{k}t^{k}$, where *X*^0^(*t*_*i*_), *Y*^0^(*t*_*i*_), *Z*^0^(*t*_*i*_) are the initial values and the *X*^*k*+1^, *Y*^*k*+1^, *Z*^*k*+1^ are obtained by applying DTM method in each subinterval as follows

$$\begin{array}{cc} X^{k+1}= & \frac{k!(\beta_{1}X_{k}-\beta_{2}X_{k}-\beta_{3}\sum_{j=0}^{k}X_{j}X_{k-j}-\beta_{4}\sum_{j=0}^{k}X_{j}Y_{k-j}-\beta_{5}\sum_{j=0}^{k}Y_{k-j}(\sum_{l=0}^{j}X_{l}Y_{j-l}))}{(k+1)!}\\ & +\frac{k!(-\beta_{6}\sum_{j=0}^{k}Z_{k-j}(\sum_{l=0}^{j}X_{l}Z_{j-l})-\beta_{7}\sum_{j=0}^{k}X_{j}Z_{k-j})}{(k+1)!}\\ Y^{k+1}= & \frac{k!(\beta_{8}Y_{k}-\beta_{9}Y_{k}-\beta_{10}\sum_{j=0}^{k}Y_{j}Y_{k-j}+\beta_{4}\sum_{j=0}^{k}X_{j}Y_{k-j}+\beta_{5}\sum_{j=0}^{k}Y_{k-j}(\sum_{l=0}^{j}X_{l}Y_{j-l}))}{(k+1)!}\\ Z^{k+1}= & \frac{k!(\beta_{11}Z_{k}-\beta_{12}Z_{k}-\beta_{13}\sum_{j=0}^{k}Z_{j}Z_{k-j}+\beta_{6}\sum_{j=0}^{k}Z_{k-j}(\sum_{l=0}^{j}X_{l}Z_{j-l})+\beta_{7}\sum_{j=0}^{k}X_{j}Z_{k-j})}{(k+1)!} \end{array}$$

for *k* = 0, 1, …, *α* − 1. The solution of each case in Table 2 is plotted in Fig 1. The solutions clarify that if there are two types of predator such that male and female feed together on same prey during mating period, the predator who has low growth rate decays fast. However, the optimal ecological scenario is case (4) because it gives a periodic solution. The case (4) denotes that case when the growth rate of prey is larger than the growth rate of predators (I) and (II).

**Table 2:** Different value of parameters.

Case	*β*_1_	*β*_2_	*β*_3_	*β*_4_	*β*_5_	*β*_6_	*β*_7_	*β*_8_	*β*_9_	*β*_10_	*β*_11_	*β*_12_	*β*_13_
(1)	0.5	0.2	0.1	0.1	0.1	0.1	0.1	0.3	0.2	0.1	0.3	0.2	0.1
(2)	0.8	0.2	0.1	0.1	0.2	0.2	0.1	0.3	0.95	0.1	0.1	0.95	0.1
(3)	0.5	0.2	0.1	0.1	0.1	0.1	0.1	0.3	0.2	0.1	0.3	0.2	0.1
(4)	0.8	0.2	0.1	0.1	0.1	0.1	0.1	0.1	0.5	0.1	0.1	0.5	0.1

## 7 Conclusion

The article is a first contribution in the literature that produces two-predator-one-prey system with the effect of *XY*^2^ term. It also solved the aforementioned mathematical model by MsDTM. The used method is fast, reliable and global convergent. The stability of the dynamic of the system was investigated. We found that the equilibrium points are asymptotically stable. The advantages of the used method have been proved in our previous work [26]. The method is more accurate than ADM, DTM, LADM and is faster than 4^th^RK. In fact that the 4^th^RK method discretizes the domain and its speed based on the step size of the discretization, but the speed of MsADM based on two factors: the length of subdomain and number of taken Taylor expansion terms. Therefore we can control the speed and accuracy of MsDTM by two factors.

The results proved that when two types of predators live in a same spot with the assumptions that male and female feed on the same prey during mating period, the ecological balance is achieved when the growth rate of prey is grater than both types of predators. Otherwise, the population of prey will decay fast and then the predator populations will decay as results of lack of food.

In future work, MsDTM can be used to solve different ODEs in the science and the presented model can be studied with fractional derivative.

## Supporting information

S1 File (BST)
